# Commentary: On the joys of perceiving: Affect as feedback for perceptual predictions

**DOI:** 10.3389/fnins.2017.00556

**Published:** 2017-10-23

**Authors:** Sabrina Trapp

**Affiliations:** ^1^Department of Psychology, Ludwig-Maximilians-University, Munich, Germany; ^2^Gonda Multidisciplinary Brain Research Center, Bar-Ilan University, Ramat-Gan, Israel

**Keywords:** predictive coding, exploration-exploitation, novelty, affect, choice, uncertainty, reinforcement (psychology)

The intuition that perception relies on prior information when inferring the causes of sensory input has received strong theoretical and empirical support (for a review see Clark, 2013). In the framework of *predictive coding*, it is assumed that that cortico-cortical feedback connections provide predictions about sensory input, and only the residual errors (prediction errors) are fed forward in the visual hierarchy to be further processed (Rao and Ballard, 1999; Lee and Mumford, 2003). It has even been suggested that conceptualizing the brain as minimizing surprise can account for several neurophysiological and neuroanatomical observations (Friston, 2005, 2010). This raises the question of why organisms are not attracted to sensory vacuums where the prediction error is zero (referred to as the ‘dark room problem’). Friston et al. (2012) argued that organisms harbor models of the environment, in which such a scenario does not exist. Changes do occur in natural environments, and consequently, the cognitive systems of organisms do expect occasional prediction errors. Leaving a sensory vacuum, such as a dark room, may translate into an attempt to seek an environment that mirrors the degree of uncertainty the organisms' mind expects. However, these frameworks are formulated on an abstract, mathematical level, leaving open the question of what motivates the organism at a *psychological* level of explanation.

A framework that has the potential to address this issue was suggested by Chetverikov and Kristjánsson (2016). Their core assumption appeals to an important psychological concept, namely affect. It is suggested that successful prediction elicits positive affect^1^. This way, affect can foster increasingly accurate predictions. Importantly, it is conjectured that affective feedback is weighted with the inverse prior probabilities of events. In other words, highly predictable information tends to elicit no positive affect. Consequently, leaving a dark room would be required in order to increase positive affect.

I conjecture that this explanatory route has something to add to the field of reinforcement learning (RL). While previous proposals have emphasized the commonalities between the process of perceptual inference and RL (Rushworth et al., 2010), the exploration-exploitation dilemma in the latter has remained untouched by the connection.

The basic tenet in RL is that organisms strive to maximize their rewards. To this end, they capitalize upon efficient learning systems that attach values to cues in the environment which lead to good decisions in the future (Sutton and Barto, 1998). However, this conjecture introduces a conflict between exploitation and exploration. Given cues that promise rewards, why should organisms explore novel information for which there is no reward history? This dilemma in RL has fostered the development of a plethora of algorithms that address this issue. For example, the *shaping bonus* suggests initializing novel information with a higher value (Kakade and Dayan, 2002). More recently, it has been suggested that novel information receives high values via generalization of known stimuli in the same environment (Gershman and Niv, 2015). However, whilst these suggestions can explain that organisms choose novel stimuli rather than ignore them altogether, they are mute on the question why organisms occasionally even prefer novel to familiar stimuli^2^. In addition, there is a puzzling effect of context. It seems that laboratory rats tend to be more neophilic, e.g., preferring novelty even to cocaine (Reichel and Bevins, 2008)^3^, whereas rats in the wild show more neophobic behavior (Barnett, 1958). As of yet, there is no overarching theory that can account both for a preference for novel to familiar stimuli and such contextual modulations.

The exploration-exploitation dilemma bears structural similarity to the dark room problem—how does novelty come into play when the organism is supposed to be driven by successful predictions? Possibly, this problem cannot be accounted for by explanations rooted in RL, but requires the reference to affect and the domain of perception. If feedback for predictions is weighted with the inverse prior probabilities of events, then laboratory rats would experience no positive affect because of the high likelihood of surrounding stimuli, and consequently, seek out for novelty.

The idea aligns with data from functional imaging: Several studies have shown that the ventral striatum, a key structure in reward coding, is also activated by mere novelty of stimulus material (e.g., Bunzeck et al., 2010). These findings suggest that novelty may be intrinsically rewarding (Wittmann et al., 2008).

Figure 1 illustrates how surprise minimization, predictive coding, affect, and reward seeking could be orchestrated. At the highest level of abstraction, one might conceptualize organisms as driven by surprise minimization, as captured in the *Free Energy Principle* (FEP) (Friston, 2005). At the lowest level of processes, i.e., perception, the FEP posits that organisms actively construct hypotheses or predictions about sensory input. Thus, the Bayesian approach logically follows from the assumption that organisms must minimize their surprise via the proxy of the free-energy. This means the “Bayesian brain” hypothesis rests on the free-energy principle, and adds a functional level of explanation, i.e., what makes the system operate in a Bayesian manner (Friston, 2010). Where does affect may come into play? Biological organisms can be assumed to strive for positive, and avoidance of negative affect (Panksepp, 2008). This motivational force may ultimately serve surprise minimization, if one assumes that positive affect can be achieved by successfully using prior information (Chetverikov and Kristjánsson, 2016).

**Figure 1:**
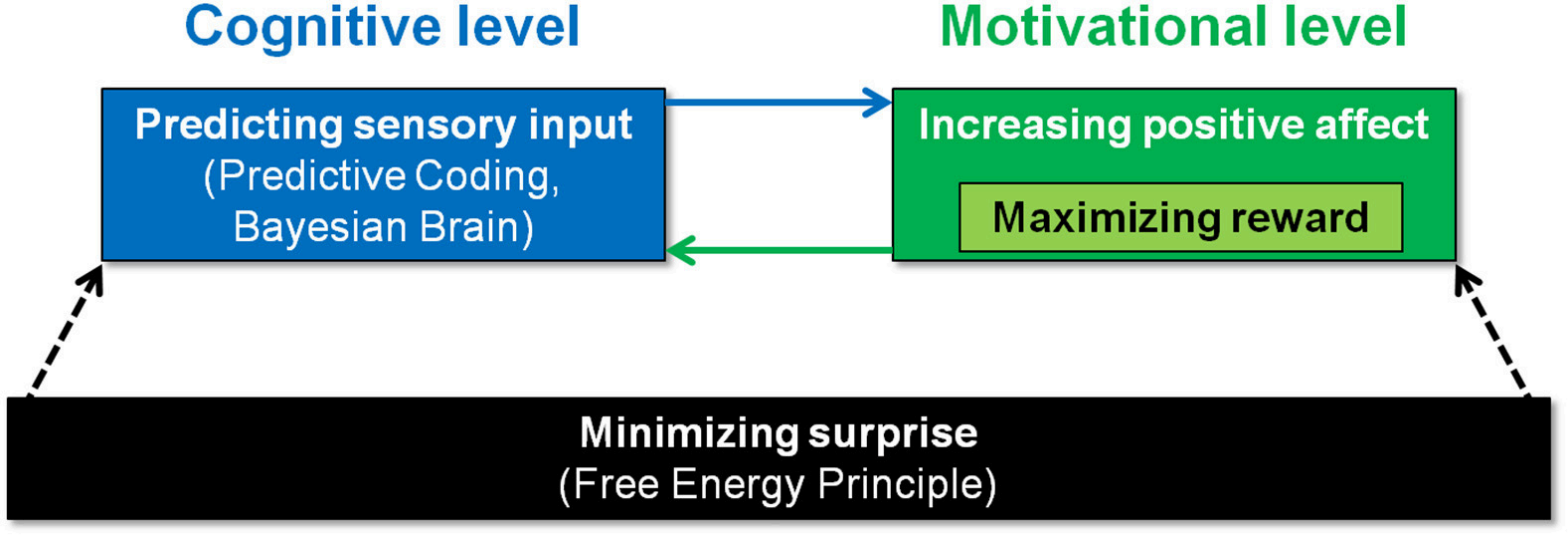
Possible goal hierarchy of biological organisms. At the bottom of the hierarchy, organisms may be driven by surprise minimization (Friston, 2010). On the level of implementation, this may require organisms (i) that apply the principles of predictive coding/Bayesian statistics (Rao and Ballard, 1999; Knill and Pouget, 2004), and (ii) that are driven by the motivation to increase positive affect. Successful predictions possibly elicit positive affect (Chetverikov and Kristjánsson, 2016). Conversely, the motivation to increase positive affect may promote prediction of sensory input.

## Author contributions

The author confirms being the sole contributor of this work and approved it for publication.

### Conflict of interest statement

The author declares that the research was conducted in the absence of any commercial or financial relationships that could be construed as a potential conflict of interest. The reviewer JM and handling Editor declared their shared affiliation.

## Footnotes

^1^The idea has already been formulated by Ramachandran and Hirstein (1999): The authors suggested that perception must be linked to limbic brain systems in order to provide incentive for discovering image correlations, binding, grouping, and identifying content.

^2^There are a few algorithms that try to address how agents make choices preferably to uncertain stimuli, e.g., by Szita and Lörincz (2008).

^3^In the predictable laboratory environment, the neophilic rats may *like* (have positive affect for) a novel stimulus enough to override their *wanting* of the highly predictable cocaine stimulus.
